# Infection of epididymal epithelial cells and leukocytes drives seminal shedding of Zika virus in a mouse model

**DOI:** 10.1371/journal.pntd.0006691

**Published:** 2018-08-02

**Authors:** Erin M. McDonald, Nisha K. Duggal, Jana M. Ritter, Aaron C. Brault

**Affiliations:** 1 Division of Vector-borne Diseases, Centers for Disease Control and Prevention, Fort Collins, Colorado, United States of America; 2 Division of High-Consequence Pathogens and Pathology, Centers for Disease Control and Prevention, Atlanta, Georgia, United States of America; University of Texas Medical Branch at Galveston, UNITED STATES

## Abstract

While primarily a mosquito-borne virus, Zika virus (ZIKV; genus *Flavivirus* in the *Flaviviridae* family) is capable of being sexually transmitted. Thirty to fifty percent of men with confirmed ZIKV infection shed ZIKV RNA in their semen, and prolonged viral RNA shedding in semen can occur for more than 6 months. The cellular reservoir of ZIKV in semen is unknown, although spermatozoa have been shown to contain ZIKV RNA and antigen. Yet, spermatozoa are not a requisite for sexual transmission, as at least one case of ZIKV sexual transmission involved a vasectomized man. To determine the cellular reservoirs of ZIKV in semen, an established animal model of sexual transmission was used. The majority of virus detected in the seminal fluid of infected mice during the peak timing of sexual transmission was from the supernatant fraction, suggesting cell-free ZIKV may be largely responsible for sexual transmission. However, some ZIKV RNA was cell-associated. In the testes and epididymides of infected mice, intracellular staining of ZIKV RNA was more pronounced in spermatogenic precursors (spermatocytes and spermatogonia) than in spermatids. Visualization of intracellular negative strand ZIKV RNA demonstrated ZIKV replication intermediates in leukocytes, immature spermatids and epididymal epithelial cells in the male urogenital tract. Epididymal epithelial cells were the principal source of negative-strand ZIKV RNA during the peak timing of sexual transmission potential, indicating these cells may be the predominant source of infectious cell-free ZIKV in seminal fluid. These data promote a more complete understanding of sexual transmission of ZIKV and will inform further model development for future studies on persistent ZIKV RNA shedding.

## Author summary

While Zika virus (ZIKV) is primarily a mosquito-borne virus, there are now confirmed sexual transmission cases of ZIKV from infected males to their partners. Using a previously established mouse model of sexual transmission, ZIKV was herein demonstrated to infect the testes and epididymides concurrently, suggesting that testicular infection is not required to seed infection of the epididymides. Also, replication of ZIKV was visualized by staining for ZIKV negative-strand RNA. ZIKV replication in leukocytes, immature spermatids and epididymal epithelial cells correlated with the peak of sexual transmission potential. Spermatozoa were rarely observed to stain positive for ZIKV replicative RNA intermediates, demonstrating spermatozoa are likely not a major source of infectious virus in semen. In addition, a greater fraction of the infectious virus in seminal fluids was cell-free versus cell-associated, suggesting that cell-free virus is responsible for sexual transmission. Taken together, these data provide strong evidence for epididymal epithelial cells and leukocytes, and not spermatozoa, as the major sources of infectious ZIKV in semen.

## Introduction

Sexual transmission of Zika virus (ZIKV; *Flaviviridae*) has been reported in thirteen countries (for a review of case reports, see [1]). ZIKV infection in males can lead to prolonged viral RNA shedding in semen, although pathological consequences of this in humans are unknown. Infectious ZIKV has been isolated from the semen of laboratory confirmed patients, including two isolations from vasectomized men, one of whom sexually transmitted ZIKV to his partner [2]. Furthermore, vasectomized men and non-vasectomized men have exhibited similar rates of ZIKV RNA shedding in semen, although RNA copy numbers were significantly lower in vasectomized versus non-vasectomized men[3]. These data strongly suggest that spermatozoa may not be the only source of sexual transmission from infected men and may not be crucial for persistent viral RNA shedding in semen.

ZIKV shedding in seminal fluids has been modeled in Interferon α/β and–γ receptor knockout AG129 mice and in Rag1-/- deficient mice treated with monoclonal antibody to the Type I Interferon Receptor (IFNAR1) [4–6]. ZIKV inoculation of immunodeficient mice and mice made transiently immunodeficient (via treatment with anti-IFNAR1) demonstrate infection of the murine male reproductive tract, specifically the testes and epididymides. Spermatogonia, primary spermatocytes, mature spermatozoa, peritubular myoid cells, and Sertoli cells stain positive for ZIKV RNA or antigen [4, 6–8]. Loss of testicular architecture, including destruction of the seminiferous epithelium has been observed following ZIKV infection [4, 5, 8]. Type I interferon receptor knockout mice (IFNAR1-/-) inoculated with contemporary Asian strains of ZIKV have exhibited atrophied seminal vesicles, reduced spermatozoa motility, and either a reduction or complete loss of mature spermatozoa [7, 9]. ZIKV antigen expression in epididymal epithelial cells, and damage to the epididymis are key features following infection [4, 8]. Furthermore, both vasectomized and non-vasectomized AG129 mice have displayed persistent shedding of viral RNA and infectious virus in seminal fluids [4], supporting findings of ZIKV infection in humans [1, 10].

The source of ZIKV and/or ZIKV RNA in semen is unknown, though spermatozoa have been suggested as a potential candidate. Previous studies of ZIKV tropism within the murine male reproductive tract identified infectious ZIKV from cellular preparations from the epididymis and ejaculates [4, 5, 8, 11]. However, cellular preparations from the epididymis or ejaculates from infected mice contain inflammatory cells, sloughed spermatogenic precursors, and potentially contaminating cell-free virus, in addition to spermatozoa. Mature spermatozoa are transcriptionally inactive, and lack endoplasmic reticula, Golgi apparati, or tRNAs, suggesting that ZIKV replication in spermatozoa is unlikely [12–15]. Thus, other cell populations that harbor replicating ZIKV are more likely to be the source(s) of infectious virus in semen.

This study was undertaken to identify cell populations within the murine male reproductive tract that could serve as a source/reservoir for ZIKV replication. To do so, ZIKV tropism for, and replication in, particular cell populations during the timing of peak sexual transmission was analyzed. A novel RNA *in situ* hybridization and sequential branched-DNA amplification assay was used to detect intracellular ZIKV RNA molecules in epididymal and testicular samples from ZIKV-inoculated mice. Probes specific to ZIKV genomic RNA (positive sense RNA; Z+) and replicative intermediates of ZIKV RNA (negative sense RNA; Z-) identified epididymal epithelial cells as a major population in the epididymis that contained replicating ZIKV during the time period associated with the peak of sexual transmission in the AG129 mouse model. Leukocyte infiltration into the testes and the epididymides was tracked during acute ZIKV infection and replicating ZIKV was identified in a fraction of these leukocytes. As previously reported, mature spermatozoa harbored genomic ZIKV RNA; however, no mature spermatozoa in the epididymis were positive for negative-strand ZIKV RNA by RNA tissue ISH. Together, these data demonstrate that epididymal epithelial cells and leukocytes are sources of replicating ZIKV and likely play a crucial role in sexual transmission of ZIKV.

## Results

### Time course analysis of the male reproductive tract after inoculation with Asian genotype ZIKV strains

**Fig 1:**
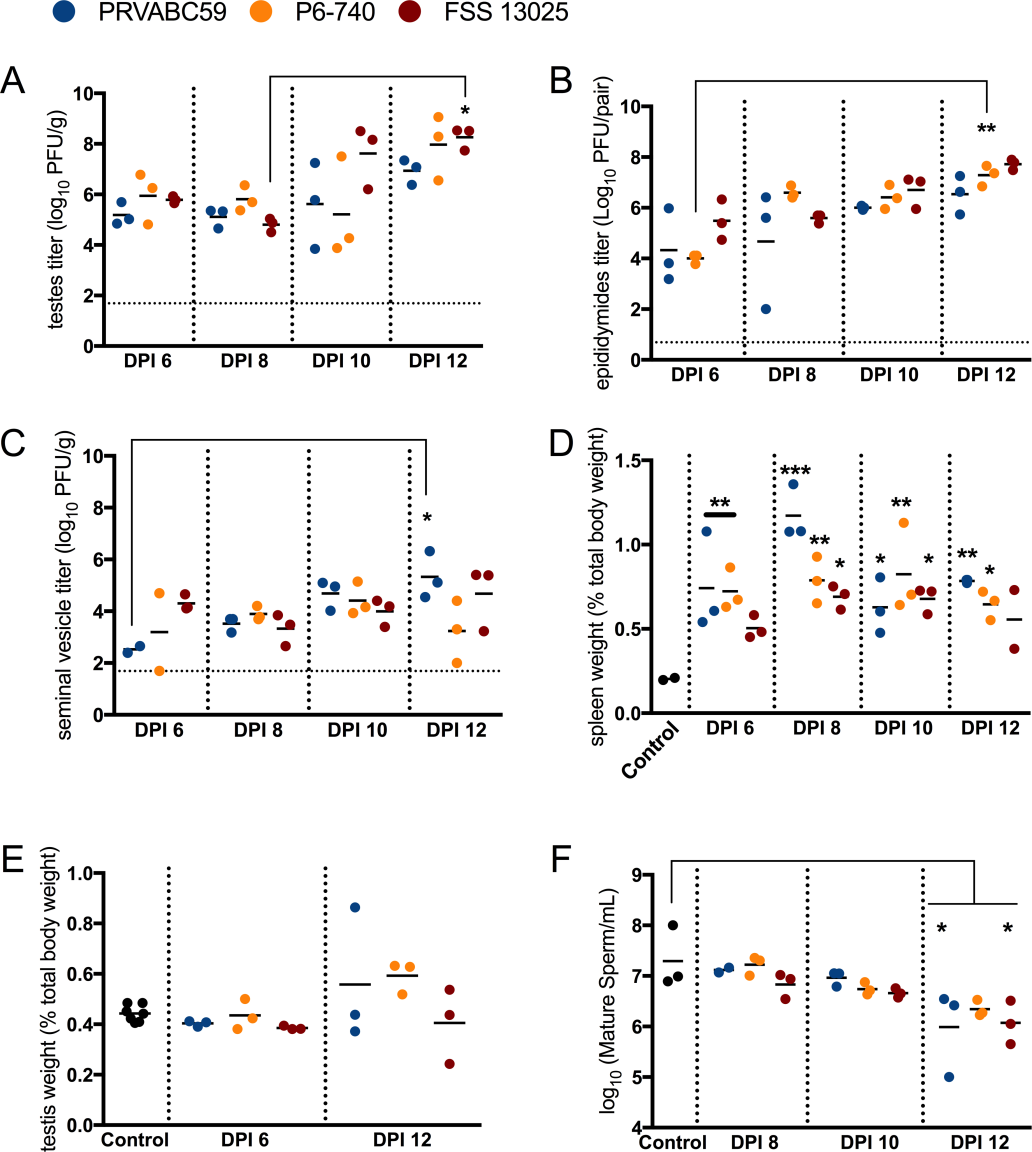
Splenomegaly and infection kinetics of the male reproductive tract during acute infection with Asian genotype ZIKV strains. Mice were inoculated s.c. with 3 log_10_ PFU of ZIKV strains PRVABC59 (blue), P6-740 (yellow), and FSS13025 (maroon) (n = 12 per virus strain). At 6, 8, 10 and 12 days post-inoculation (dpi), three mice from each group were euthanized. (A) Viral titers in the testis. (B) Viral titers in the epididymis. The dashed line represents the limit of detection for the plaque assay. (C) Viral titers in the seminal vesicles. (D) Spleen weights were recorded and the ratio of (spleen to body weight) x 100 was measured. (E) Testicular weight as a percentage of total body weight. (F) Spermatozoa concentration decreased during acute infection. Epididymides were minced in warm PBS and a portion of the resulting cell suspension was stained with trypan blue and counted on a hemocytometer. Each symbol represents one mouse. P-values were determined by 2way ANOVA. *, p<0.05, ** p<0.01, ***p < 0.0001.

Previous published accounts demonstrated that AG129 mice inoculated with different Asian genotype ZIKV strains shed ZIKV in seminal fluids at similar rates and infectious viral titers during the second week post-infection, a time period that corresponded with peak efficiency for sexual transmission [4, 5]. To assess the kinetics of ZIKV infection of the male reproductive tract, AG129 mice were inoculated with 3 log_10_ PFU of three different Asian genotype ZIKV strains (PRVABC59, P6-740 or FSS 13025), and three mice per group were euthanized at days 6, 8, 10 and 12 post-inoculation. Infectious virus was present in the testes, epididymides, and seminal vesicles of ZIKV-inoculated mice at all time points assessed, but the titers for each organ did not drastically increase over time (Fig 1A–1C). The highest titers in the testes were observed on dpi 12, and the mean peak titer for PRVABC59-, P6-740- and FSS 13025-inoculated mice ranged from approximately 7 to 8.3 log_10_ PFU/gram tissue (Fig 1A; FSS 13025 dpi 8 vs 12 p<0.05). Infectious viral titers were calculated per pair of epididymides, and were highest on dpi 12, with a range of 6.5 to 7.7 log_10_ PFU/pair of epididymides (Fig 1B; P6-740 dpi 6 vs 12 p<0.01). Seminal vesicle titers were highest from dpi 10 to dpi 12 and ranged from 4.4 to 5.3 log_10_ PFU/gram tissue (Fig 1C; PRVABC59 dpi 6 vs 12 p<0.05).

Splenomegaly was observed for all the ZIKV-inoculated mice (Fig 1D). Significant increases in spleen weight (as a function of initial weight of the mouse) were observed in PRVABC59- and P6-740-inoculated mice as early as dpi 6 (PRVABC59 and P6-740 vs control p<0.01). The greatest mean proportional increase in spleen mass occurred in all mice by dpi 8 (Fig 1D; FSS 13025 vs control p<0.05, P6-740 vs control p<0.01, PRVABC59 vs control p<0.0001). Splenomegaly was observed through dpi 12. Testes weight was also measured during acute infection. From dpi 6 to dpi 12, there was a trend for increased testis weight in the ZIKV-inoculated mice (Fig 1E; *ns*, p values ranged from 0.2 to 0.9).

Others have noted testicular pathological features in ZIKV-infected mice. To determine if spermatogenesis was impaired in ZIKV-inoculated AG129 mice, sperm counts were performed at dpi 8, 10 and 12. All ZIKV-inoculated mice had decreased spermatozoa counts from dpi 8 to 12, with a significant decrease on dpi 12 observed for PRVABC59- and FSS 13025-inoculated mice, compared to age-matched control mice (Fig 1F; spermatozoa counts from control mice were 7.3 log_10_ spermatozoa/mL vs. 6 log_10_ spermatozoa/mL (PRVABC59) and 6.1 log_10_ spermatozoa/mL (FSS 13205); p<0.05). P6-740-inoculated mice had lower mean spermatozoa counts over time, but a statistically significant decrease in mean spermatozoa counts was not observed by dpi 12 (6.3 log_10_ spermatozoa/mL, p = 0.9). Thus, during acute infection of AG129 mice with ZIKV, infection may result in decreased spermatozoa counts.

### Mature spermatozoa contain ZIKV genomic RNA

Spermatozoa have been posited as a potential source of infectious ZIKV. Using confocal microscopy, the fraction of spermatozoa containing ZIKV genomic RNA was calculated. Epididymal cell suspensions from ZIKV-inoculated mice during the timing of peak sexual transmission potential (dpi 10) were stained with probes specific to ZIKV genomic RNA (positive sense RNA; Z+). The percent of Z+ mature spermatozoa ranged from 3.8% to 30% (Table 1).

**Table 1:** ZIKV RNA (+) strand staining of mature spermatozoa from Asian genotype ZIKV-inoculated mice.

ZIKV strains	N	% ZIKV (+), N	Mean % ZIKV (+), SD
PRVABC59	50	14%, 7	8.9%, 7.2
	106	3.8%, 4	
P6-740	60	5%, 3	8.3%, 4.7
	60	11.7%, 7	
FSS 13025	50	30%, 15	18.3%, 16.5
	30	6.7%, 2	

N is the number of mature spermatozoa; each row is a biological replicate

Next, it was determined whether mature spermatozoa contained replicative intermediates of ZIKV RNA on dpi 10. To do this, probe sets specific to replicative intermediates of ZIKV RNA (negative sense RNA; Z-) were used. An average of 4.7% of mature spermatozoa stained positive for Z-, which was ~4-fold lower compared to spermatozoa that stained for Z+ (Table 2). The staining for Z+ and Z- was limited in spermatozoa: a range of 1–4 small foci of staining was seen per spermatozoa. This was in stark contrast to the multiple large foci of staining present in round cells (leukocytes and/or sloughed spermatogenic precursors) from the same preparation (S1 Fig). This prompted closer inspection of the male reproductive tract to identify cell populations that could contribute to sexual transmission.

**Table 2:** ZIKV RNA (+) and (-) strand staining of mature spermatozoa from PRVABC59-inoculated mice.

N	% ZIKV (+), N	% ZIKV (-), N	% Co-stained, N	Mean % ZIKV (+), SD	Mean % ZIKV (-), SD
78	17.9%, 14	1.3%, 1	0%, 0	21.3%, 4.7	4.7%, 4.9
61	24.6%, 15	8.2%, 5	4.9%, 3		

N is the number of mature spermatozoa; each row is a biological replicate

### RNA tissue ISH identifies epididymal epithelial cells as the major cell type containing replicative intermediates of ZIKV

**Fig 2:**
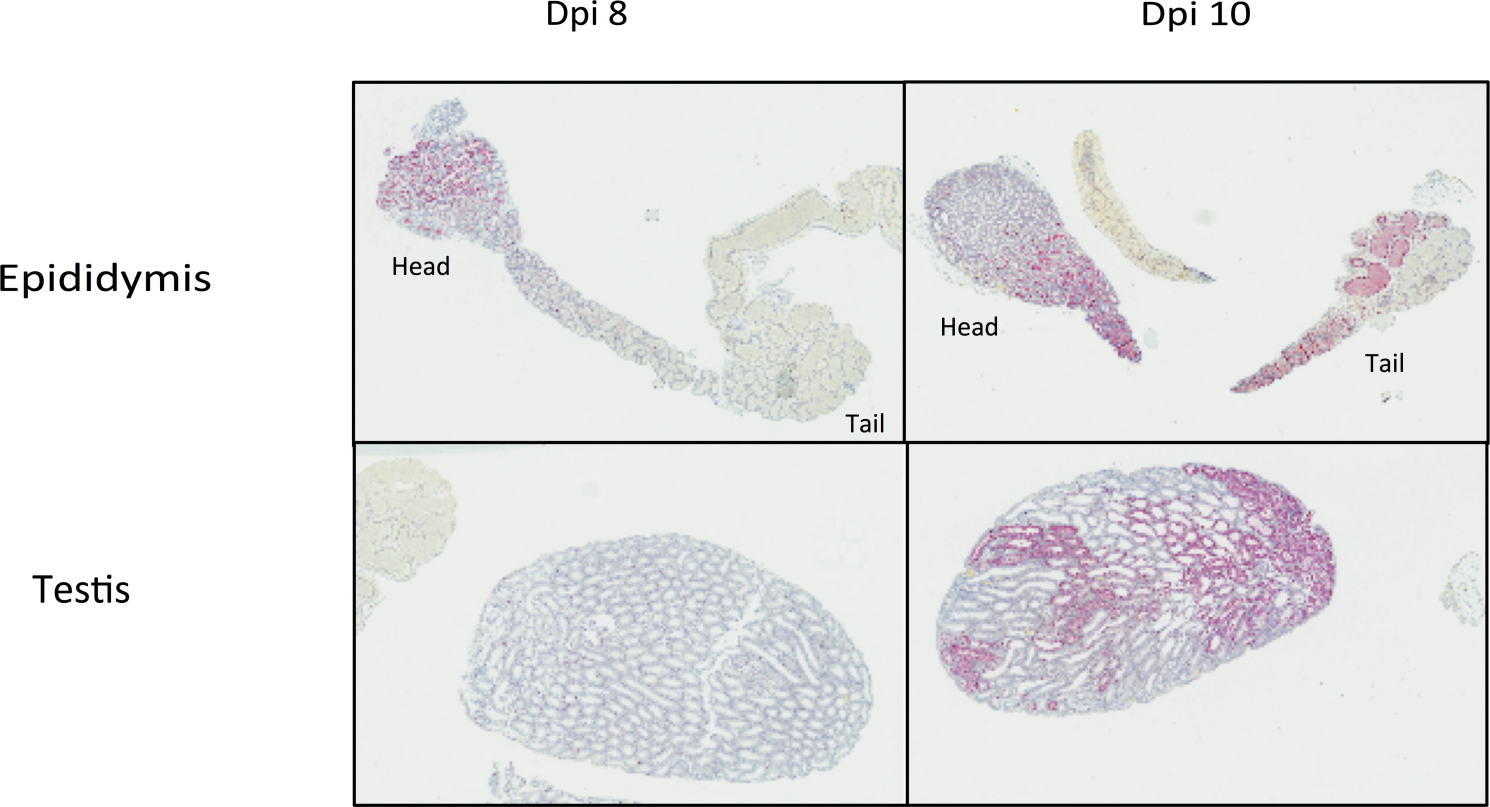
ZIKV genomic RNA localization in PRVABC59-inoculated mouse epididymis and testis at dpi 8 and 10. At dpi 8, ZIKV RNA is localized to the head of the epididymis, and not visible in the testis at this magnification. At dpi 10, ISH signal is decreased in the head of the epididymis and strong in the body and tail of the epididymis, and within seminiferous tubules of the testis. ISH, Z (+) strand, hematoxylin counterstain of nuclei, Original magnification: 7X.

As it was crucial to identify cell populations that harbored replicating ZIKV, testes and epididymides from PRVABC59-inoculated mice were evaluated by histology and tissue *in situ* hybridization (*IS*H) for both ZIKV genomic and replicative RNA at dpi 8 (n = 3) and dpi 10 (n = 3). Overall, ZIKV RNA was concentrated in the head of the epididymis, and minimally present in the testis, at dpi 8. By dpi 10, there was decreased staining in the epididymal head, with extensive staining in the body and tail of the epididymis, and extensive, patchy staining within seminiferous tubules of the testis (Fig 2).

**Fig 3:**
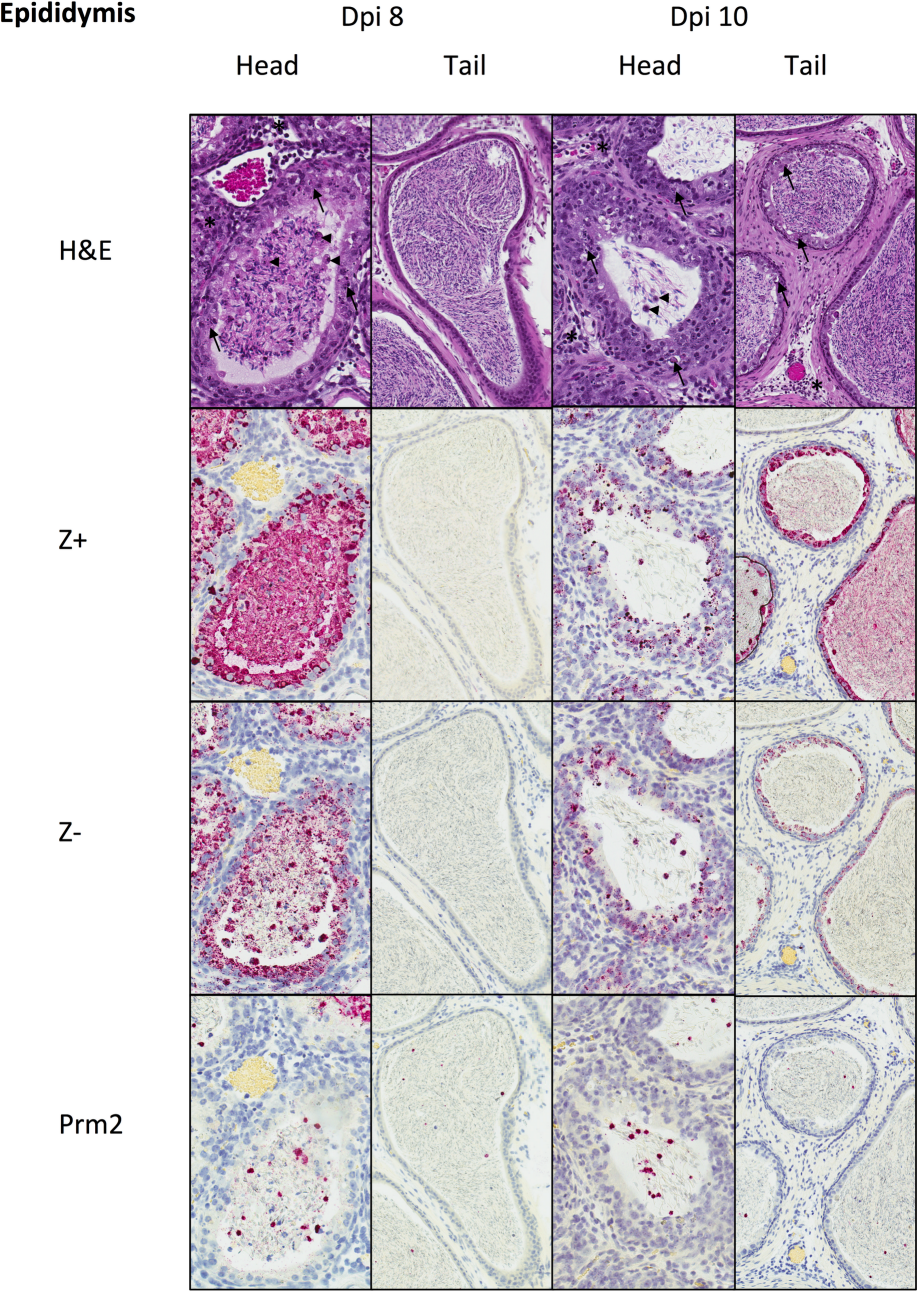
Histopathology and tissue ISH in PRVABC59-inoculated mouse epididymis at dpi 8 and 10. At dpi 8, the head of the epididymis shows epithelial necrosis (arrows) and mild interstitial inflammation (*). Tubular lumens contain spermatozoa and degenerating round cells (arrowheads). The tail of the epididymis showed no epithelial changes and contained mature spermatozoa. RNA genomic RNA (Z+) and replicative intermediates (Z-) were present within epididymal epithelium and luminal immature spermatozoa and round cells in the head of the epididymis, but not in epithelium or mature spermatozoa in the tail of the epididymis. Many intraluminal round cells were Prm2+ spermatids. At dpi 10, epithelial necrosis and inflammation were similar in the head of the epididymis as seen at dpi 8, and these changes were also seen in the tail of the epididymis. ZIKV genomic RNA (Z+) was decreased in the epithelium and luminal cells of the head of the epididymis, and was also extensively present in the epithelium and spermatozoa of the tail of the epididymis. ZIKV replicative intermediates (Z-) were also present in the epididymal epithelium and in luminal round cells of both the head and the tail, but not in mature spermatozoa of the tail. Many intraluminal round cells were Prm2+ spermatids. H&E and ISH with hematoxylin counterstain of nuclei, Original magnifications: 400x (head), 200X (tail).

At dpi 8, the head of the epididymis had multifocal necrosis of epididymal epithelial cells (EEC) and mild, mixed, neutrophilic and mononuclear interstitial inflammation. Tubular lumens contained spermatozoa and degenerating round cells (sloughed spermatogenic precursors and/or leukocytes). The tail of the epididymis was without epithelial alterations and contained mature spermatozoa. RNA ISH detected ZIKV genomic RNA extensively within intact and necrotic EEC, intraluminal round cells, and immature spermatids in the head of the epididymis. ISH for ZIKV replicative RNA showed a similar distribution, with lesser amounts of staining than genomic RNA. Neither genomic nor replicative RNA was detected in the tail of the epididymis at dpi 8. ISH was also performed for protamine-2 (PRM2) mRNA, which encodes for a chromatin protein that replaces histones and is specifically expressed in only step 7 round spermatids through step 15 elongated spermatids [16]. ISH for Prm2 identified many of the luminal round cells as sloughed round spermatids (Fig 3). At dpi 10, epididymal epithelial necrosis and mild inflammatory changes extended beyond the head of the epididymis and into the body and tail. ZIKV genomic and replicative RNA were both detected with a similar distribution, but to a lesser extent than at dpi 8, within the epididymal head, and were also detected extensively in the epididymal epithelium of the body and tail. Mature spermatozoa and luminal round cells, including spermatids, were positive for ZIKV genomic RNA, but staining of mature spermatozoa for negative strand ZIKV RNA was not observed (Fig 3).

**Fig 4:**
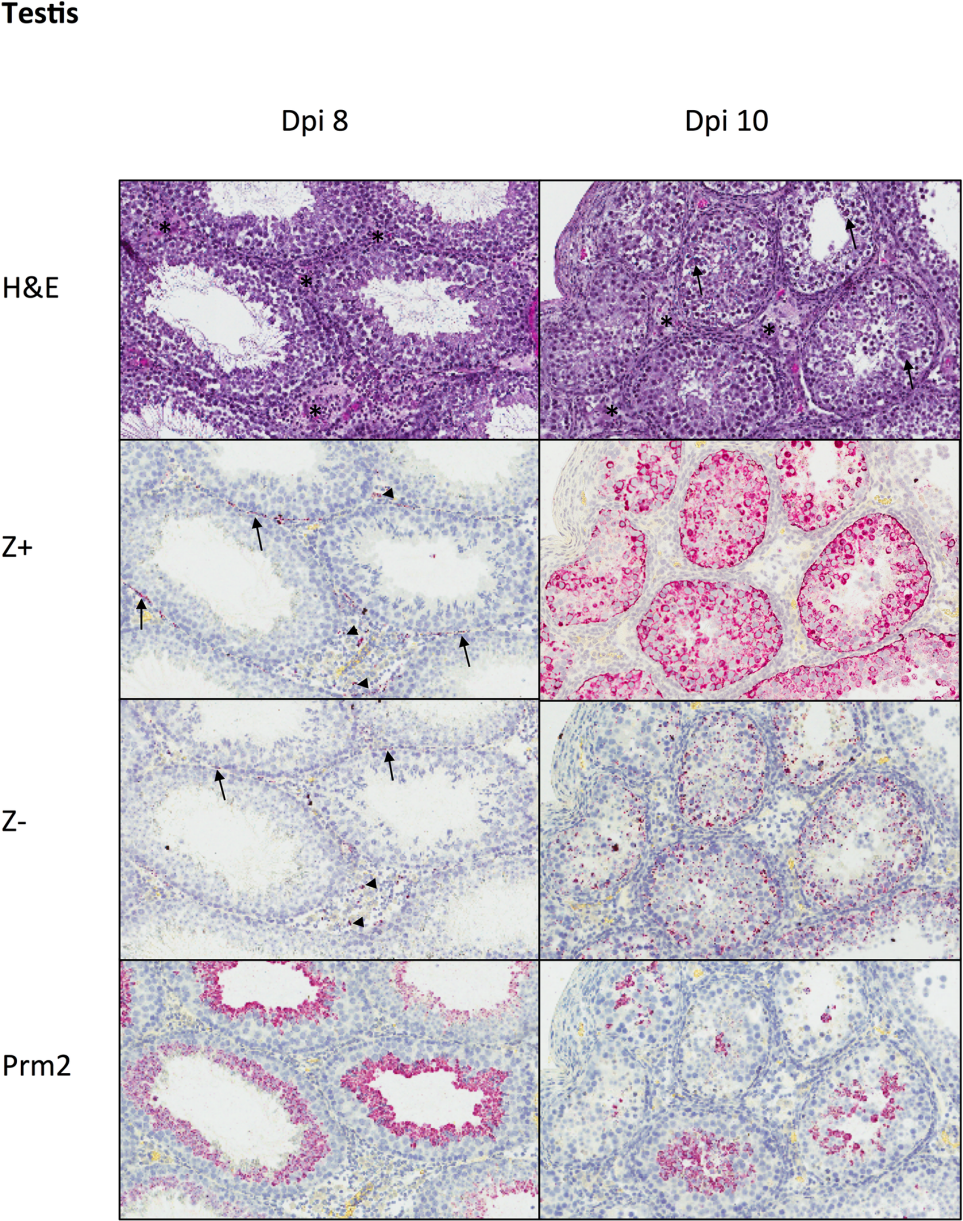
Histopathology and tissue ISH in PRVABC59-inoculated mouse testis at dpi 8 and 10. At dpi 8, testis showed mild interstitial inflammation (*), without alterations in seminiferous epithelium. ZIKV genomic RNA (Z+) and replicative intermediates (Z-) were localized to scattered interstitial leukocytes (arrowheads) and peritubular myoid cells (arrows). Prm2+ staining showed round spermatids along the luminal surface of seminiferous tubules. At dpi 10, mild interstitial inflammation (*) was accompanied by patchy degeneration of seminiferous epithelium (arrows). Zika virus genomic RNA (Z+) and replicative intermediates (Z-) were present within all layers of seminiferous epithelium, and there was a reduction in the number of Prm2+ round spermatids compared to dpi 8. H&E and ISH with hematoxylin counterstain of nuclei, Original magnifications: 200X.

Testis exhibited mild neutrophilic to mixed interstitial inflammation and edema at dpi 8 and 10 (Fig 4). Seminiferous tubules were unaffected at dpi 8, but at dpi 10, 2 of 3 mice had focal degeneration and necrosis of seminiferous epithelium, with decreased spermatozoa formation. At dpi 8, identification of ZIKV genomic RNA was rare and localized to scattered interstitial leukocytes, peritubular myoid cells, and within the testicular capsule; there was no staining of seminiferous epithelium. By dpi 10, extensive ZIKV genomic RNA staining within all layers of epithelium of some seminiferous tubules was evident. As in the epididymis, ZIKV replicative RNA detection mirrored genomic RNA distribution, but with lesser overall amounts of staining. ISH for Prm2 showed a decrease in number of round spermatids within seminiferous tubules at dpi 10 compared to dpi 8 (Fig 4).

### Leukocytes in the testes and epididymal lumen contain replicating ZIKV

**Fig 5:**
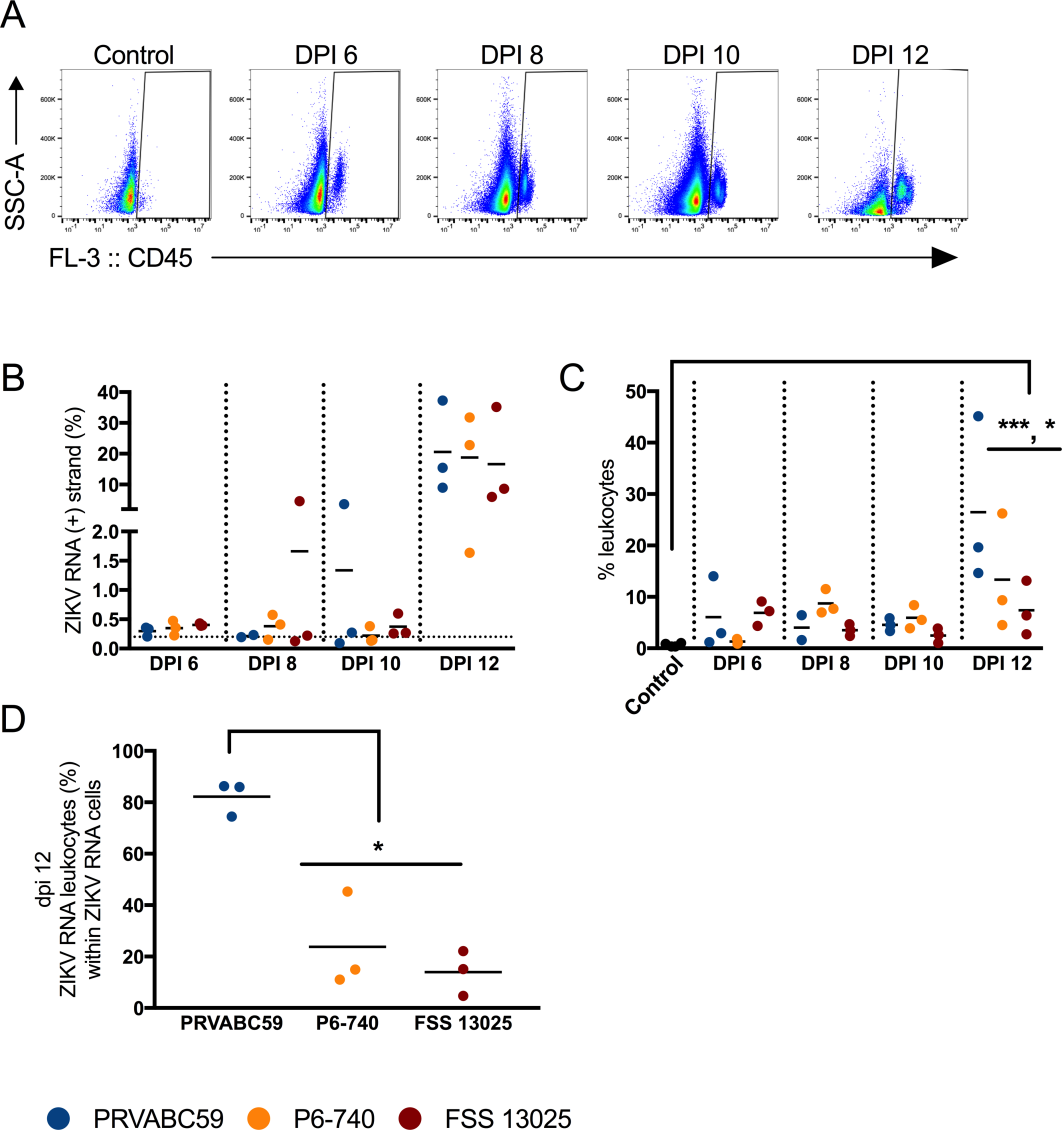
Testicular leukocytes contained genomic ZIKV RNA. Mice were inoculated s.c. with 3 log_10_ PFU of ZIKV strains PRVABC59 (blue symbols), P6-740 (yellow), and FSS13025 (maroon) (n = 12 per virus strain). At six, eight, ten and twelve dpi, three mice from each group were euthanized. A single cell suspension of each testis was stained according to the PrimeFlow assay. (A) Representative dot plots showing testicular cells from uninfected control mice and ZIKV-infected mice stained with anti-CD45 antibody. (B) Percentage of ZIKV RNA (+) strand cells. Uninfected control mice were used to set gates for ZIKV RNA+ cells (gates were set such that 0.2% or less of the total population in the uninfected control was found in the ZIKV RNA+ gate.). (C) CD45+ leukocyte infiltration into the testes over time. (D) Percentage of ZIKV RNA (+) strand leukocytes within ZIKV RNA (+) strand cells. Each symbol represents one mouse. P-values were determined by 2way ANOVA. *, p<0.05, *** p<0.0001.

Taking into account that pathology showed a mixed neutrophilic and mononuclear interstitial inflammation in the epididymis and testes, infection induced leukocyte infiltration was monitored by flow cytometry. Testes samples from mice sacrificed at 6, 8, 10, and 12 dpi were co-stained with an antibody to the pan-leukocyte marker, CD45, and with Z+ probes (see Fig 5A for representative gating scheme). In the testes, there was an increase in the fraction of leukocytes by dpi 6 (only 0.6% of testicular cells were leukocytes in negative control mice) and this increase was sustained through dpi 12. By dpi 12, PRVABC59-inoculated mice showed the greatest increase in leukocytes, with 26.5% of the total testicular cells staining positive for CD45 (Fig 5C; p < 0.0001 for PRVABC59; p < 0.05 for P6-740). Z+ cells could be robustly detected in the testes on dpi 12 (Fig 5B), and 82.2% of the Z+ cells co-stained with CD45 in the PRVABC59-inoculated mice. This was significantly greater than that same population in the P6-740- and FSS 13025-inoculated mice (23.8% and 14%, respectively; p < 0.05) (Fig 5D).

**Fig 6:**
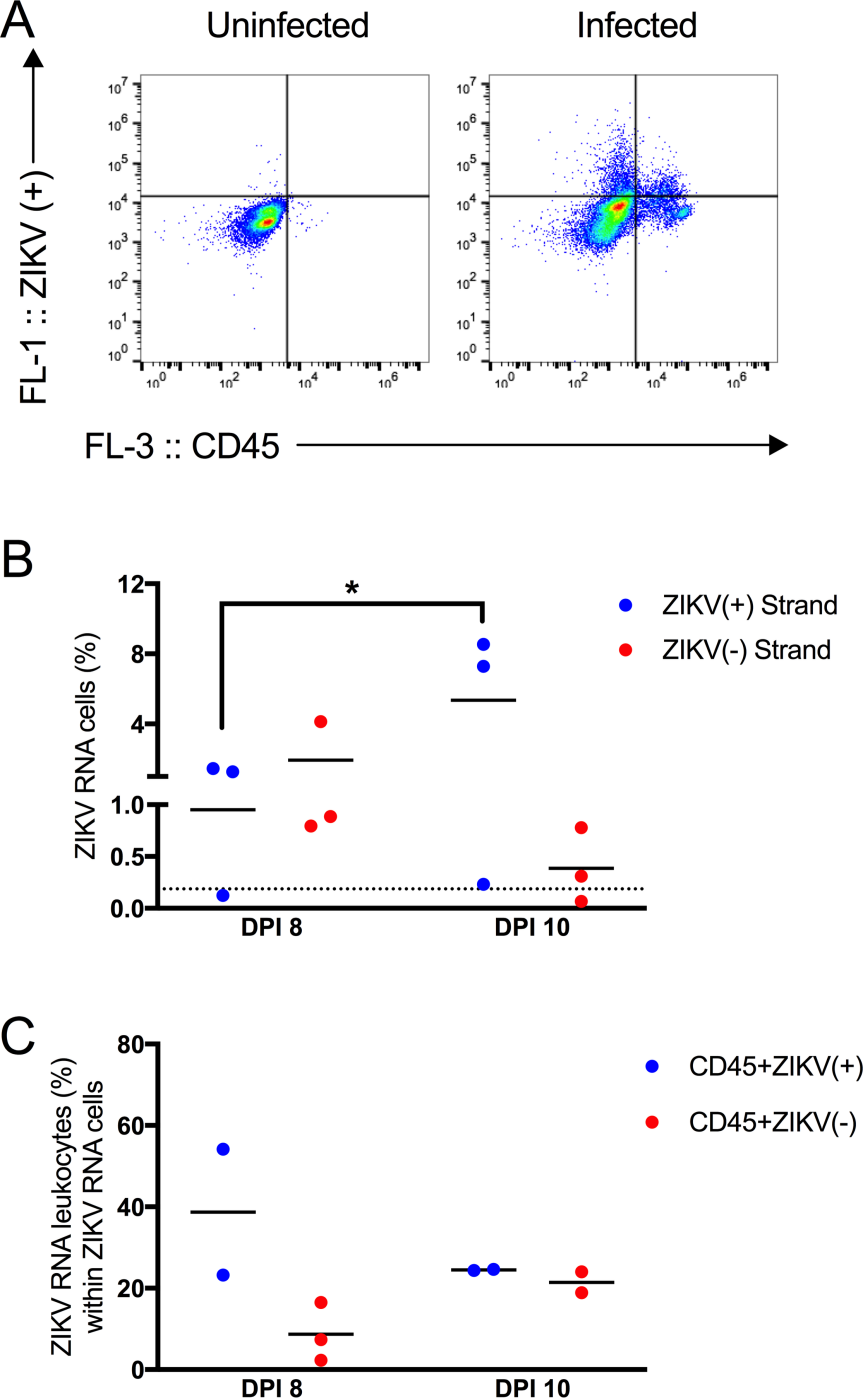
Testicular leukocytes contained replicating ZIKV RNA. (A) Representative dot plots showing testicular cells stained with ZIKV RNA (+) strand probes (y-axis) and anti-CD45 antibody from an uninfected or infected mouse (x-axis). (B) Percentage of ZIKV RNA (+) or (-) strand cells. On dpi 8, none of the cells stained for ZIKV RNA (-) strand above background. On dpi 10, two of the three mice contained cells in the epididymal lumen that stained for ZIKV RNA (-) strand above background. (C) Percentage of ZIKV RNA leukocytes within ZIKV RNA cells. Each symbol represents one mouse. P-values were determined by 2way ANOVA. *, p<0.05.

**Fig 7:**
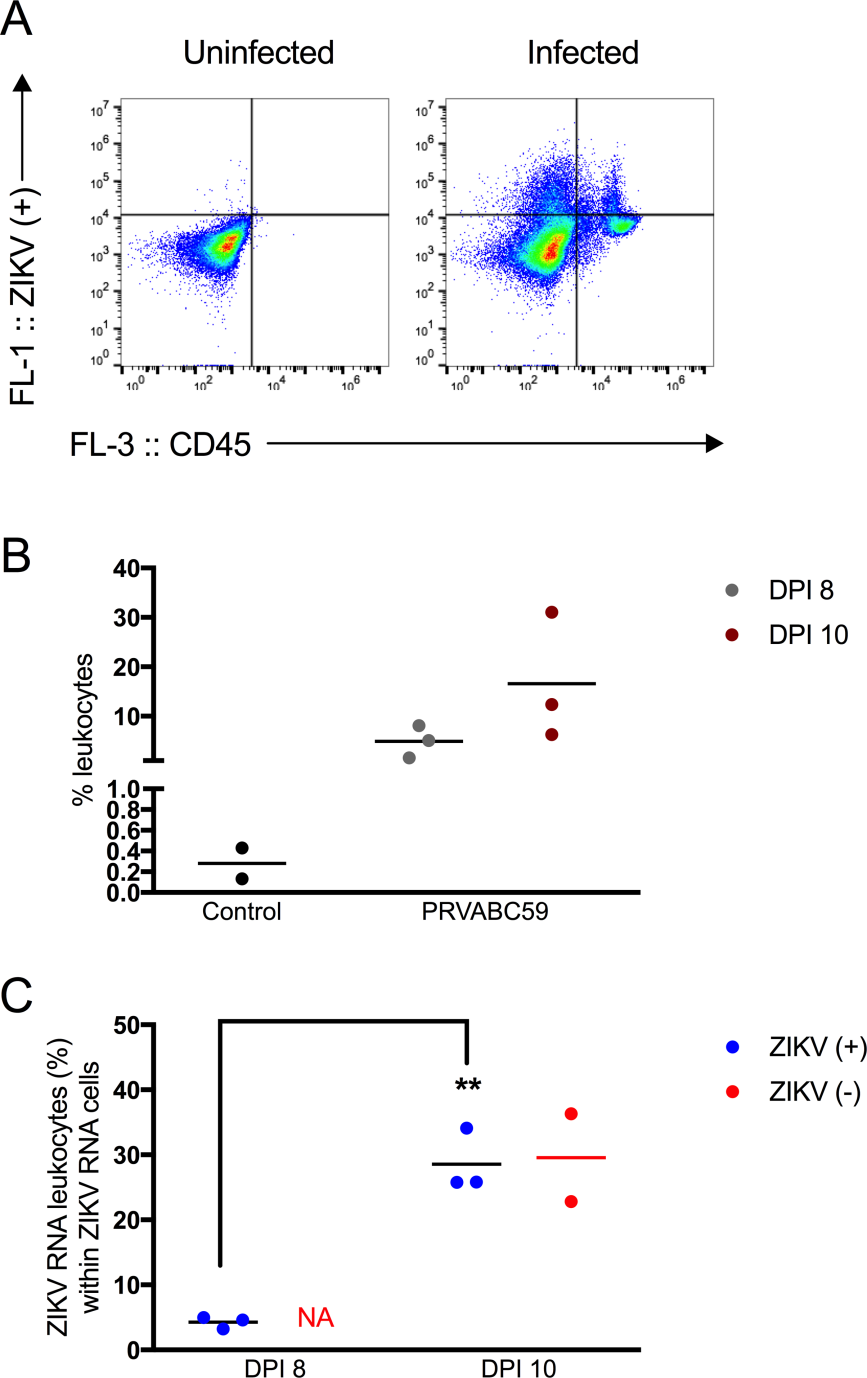
Leukocytes in the epididymal lumen contained replicating ZIKV RNA. (A) Representative dot plots showing cells from the epididymal lumen stained with ZIKV RNA (+) strand probes (y-axis) and anti-CD45 antibody (x-axis) from an uninfected or infected mouse. (B) Percentage of CD45+ leukocytes in control and infected animals. Leukocytes increase in the epididymal lumen upon ZIKV infection (black dots represent leukocytes from control animals; gray dots represent leukocyte from PRVABC59-inoculated mice on dpi 8 and maroon dots represent leukocytes from PRVABC59-inoculated mice on dpi 10). (C) Percentage of ZIKV RNA leukocytes within ZIKV RNA cells. On dpi 8, none of the cells stained for ZIKV RNA (-) strand above background. On dpi 10, two of the three mice contained cells in the epididymal lumen that stained for ZIKV RNA (-) strand above background. Each symbol represents one mouse. For the (+) strand, p-values were determined by unpaired t-test. *, p<0.05, ** p<0.01.

Leukocytes with actively replicating ZIKV could be one mechanism through which ZIKV is trafficked to the testis or epididymal lumen. In the testes, leukocytes containing Z- were identified on both 8 and 10 dpi (Fig 6B). Leukocytes had been previously observed in the epididymal lumen during the peak time period for sexual transmission [4]. At dpi 8 and dpi 10, leukocytes comprised 4.9 and 16.6% of the epididymal lumen cells, respectively (Fig 7A and 7B). This was in stark contrast to the control mice, in which only 0.3% of the epididymal lumen cells were leukocytes. The fraction of Z+ cells in the epididymal lumen increased significantly from 8 to 10 dpi (p < 0.05; Fig 7C), and there was an approximately 6.7-fold increase of Z+ cells that were leukocytes (4.3% vs. 28.6%) between the two time points (Fig 7C; p < 0.001). At dpi 8, Z- RNA cells could not be detected above background by flow cytometric methods. At dpi 10, epididymal lumen cell suspensions from two of the three mice exhibited staining for Z-, with 29.6% of the Z- cells being leukocytes (Fig 7C).

**Fig 8:**
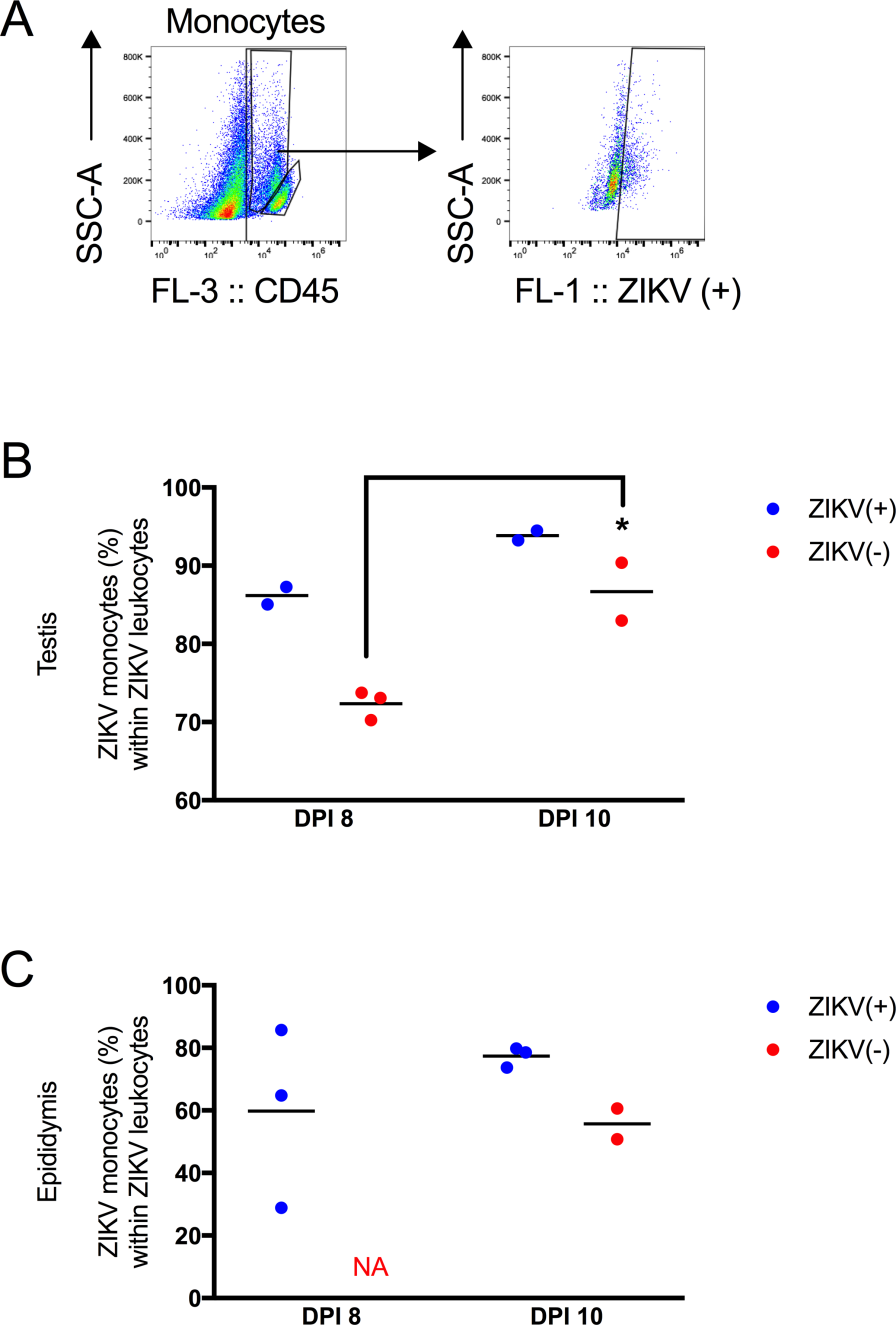
Monocytes were the major leukocyte population that contained ZIKV. (A) Representative gating scheme. Monocytes are gated (high SSC in the CD45+ population) and then the ZIKV (+) RNA gate is applied to this population. (B) Percentage of ZIKV RNA monocytes out of ZIKV RNA CD45+ leukocytes in the testis. (C) Percentage of ZIKV RNA monocytes within ZIKV RNA leukocytes in the epididymis. Each symbol represents one mouse. P-values were determined by 2way ANOVA. *, p<0.05.

Since recent work has implicated circulating monocytes as a reservoir for ZIKV, Z+ and Z- leukocytes were gated to distinguish monocytes from lymphocytes. As shown in Fig 8, the side scatter (SSC) parameter was used to separate monocytes from lymphocytes in the CD45+ population (Fig 8A). The lymphocyte population contained few Z+ and Z- cells and was not analyzed further. In the testes, the fraction of leukocytes that were monocytes staining positive for Z- significantly increased from 8 to 10 dpi (Fig 8B, p < 0.05). In the epididymal lumen, 55.7% of the Z- leukocytes were monocytes on dpi 10 (Fig 8C). These data suggest monocytes could both serve as a site of replication and for trafficking ZIKV to immunologically privileged sites.

### Immature spermatids contain replicating ZIKV

**Fig 9:**
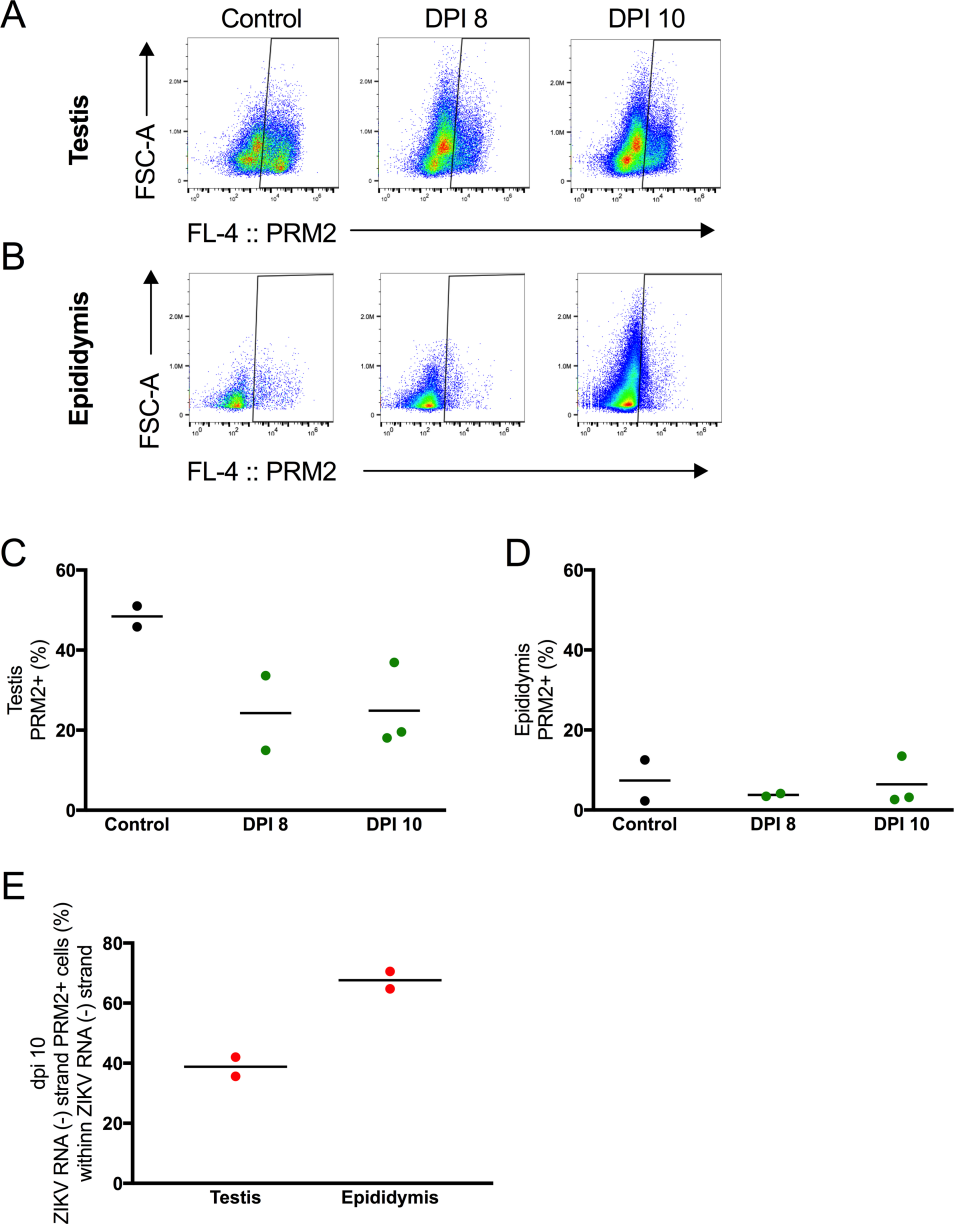
Immature spermatids contained genomic and replicative ZIKV RNA. (A) Representative dot plots showing testicular cells from 8 and 10 dpi stained with probes to PRM2 mRNA. (B) Representative dot plots showing epididymal lumen cells from 8 and 10 dpi stained with probes to PRM2 mRNA. (C) Percentage of PRM2+ immature spermatids in the testis. (D) Percentage of PRM2+ immature spermatids in the epididymal lumen. (E) Percentage of ZIKV (-) strand RNA cells that co-stained with PRM2 in the testis and epididymal lumen. Each symbol represents one mouse.

To address whether the decrease in mature spermatozoa from 8 to 10 dpi was associated with a decrease in spermatids undergoing spermatogenesis, immature spermatids were quantified by flow cytometry. In the testes, ZIKV infection led to approximately a 50% decrease in PRM2+ round and elongating spermatids (Fig 9A and 9C). Immature spermatids were also detected in the epididymal lumen (Fig 9D). On dpi 10, thirty-nine and 68% of Z- cells co-stained with PRM2 in the testis and epididymal lumen respectively (Fig 9E). These data, combined with the above, demonstrated that transcriptionally active cells, such as leukocytes and PRM2+ spermatids, contributed a large proportion of ZIKV replication in the testes and epididymides.

### The majority of infectious virus in ejaculates was not cell-associated

**Fig 10:**
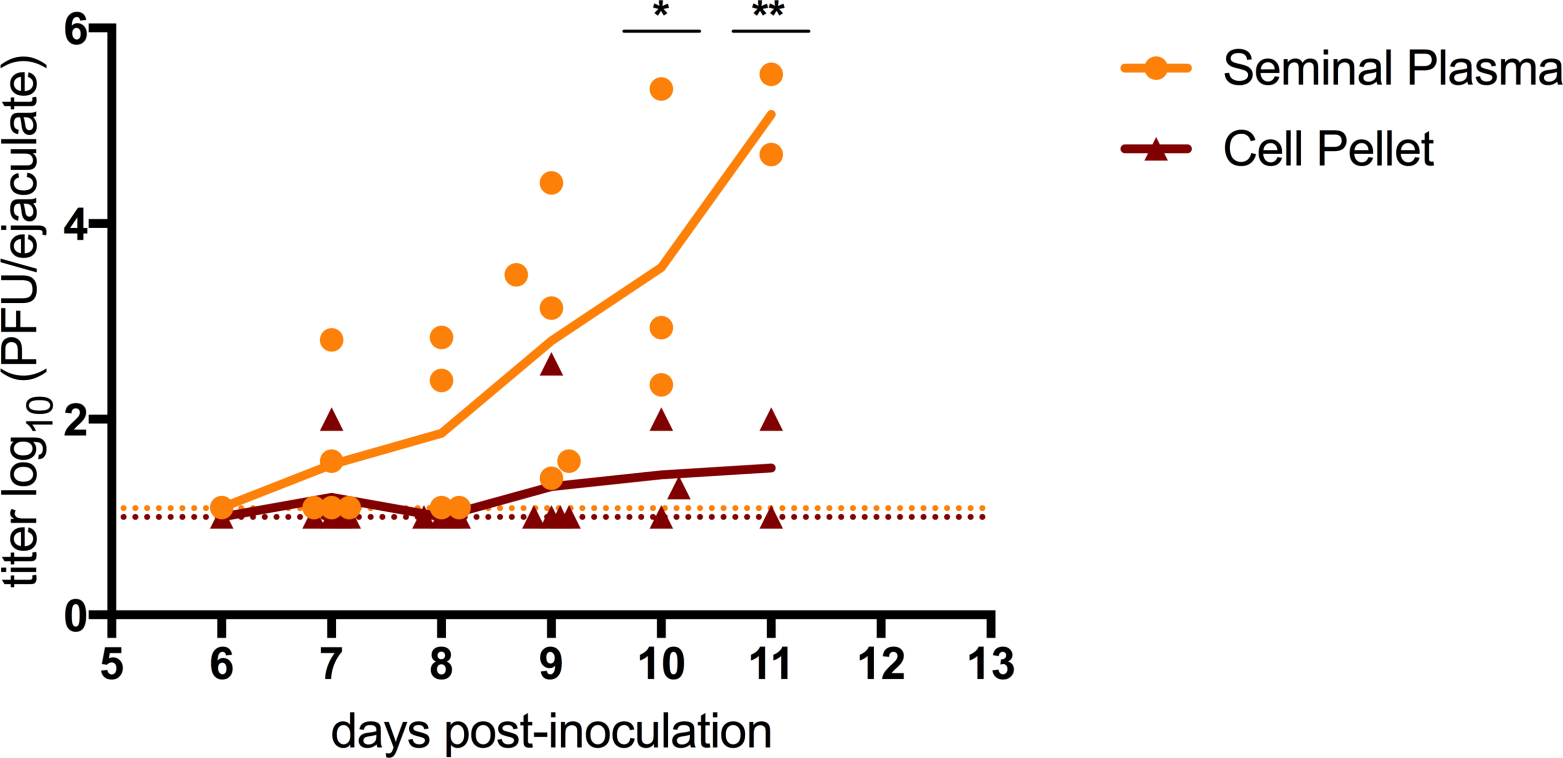
Infectious ZIKV in seminal plasma versus cellular fractions from seminal fluids. Mice were inoculated s.c. with 3 log_10_ PFU of ZIKV strain PRVABC59. Viral titers in seminal fluids collected from mice dpi 6 to 12. Individual ejaculates (n = 21) were partitioned into a cell pellet (maroon triangle) and a filtered supernatant (yellow circle). The colored lines represent the mean values across all mice and are used to make trends more visible. The limits of detection are represented by dashed lines (1.0 log_10_ for the cell pellet and 1.09 log_10_ for the filtered ejaculates). P-values were determined by 2way ANOVA. *, p<0.05; ** p<0.01.

ZIKV genomic RNA and replicating ZIKV was detected within intact and necrotic EEC, and in leukocytes and spermatogenic precursors in epididymal lumens. To address whether infectious ZIKV in seminal fluids originated from ZIKV-infected cells or was cell-free infectious virus, ejaculates were collected from PRVABC59-inoculated mice and divided into cell pellet and seminal plasma fractions. Of the collected ejaculates, 15 of 21 (71%) were positive for infectious virus. Of the 15 ejaculates positive for infectious virus, 60% (9 ejaculates) had infectious virus only in the seminal plasma (and not in the cell fraction). All of the ejaculates with infectious virus in the cell pellet also contained infectious virus in the seminal plasma (29% of the total ejaculates). In addition, the filtered seminal plasma contained higher titers of infectious virus. On dpi 10 and 11, the mean titer of infectious virus in the filtered seminal plasma (3.6 and 5.1 log_10_ PFU/ejaculate) was significantly higher than the mean titer in the cell pellet (1.4 and 1.5 log_10_ PFU/ejaculate) (Fig 10; p<0.05, p<0.01). This trend was observed during the peak of sexual transmission potential, beginning on 7 to 11 dpi. Thus, infectious ZIKV in seminal fluids was both cell-free and cell-associated, with some ejaculates containing only cell-free infectious ZIKV, and higher viral titers found in the filtered seminal plasma at later time points.

## Discussion

In this study, a highly sensitive *in situ* hybridization assay was used to identify host cell populations in the mouse male reproductive tract that contained replication intermediates of ZIKV prior to and during the peak timing of sexual transmission (dpi 8 and 10, respectively). By tissue ISH, there was more intense staining for ZIKV RNA in the epididymis as compared to the testes at dpi 8. The more intense staining for ZIKV RNA in the head of the epididymis suggests that the testes and epididymis could be infected concurrently. In the testes, ZIKV positive and negative strand RNA was identified within interstitial leukocytes, peritubular myoid cells and immature spermatids. In the epididymis, ZIKV positive and negative strand RNA staining was found extensively in EEC. In the epididymal tubular lumens, replicating ZIKV was identified in leukocytes and immature spermatids. Consistent with other reports, 8–18% of mature spermatozoa from epididymal cell suspensions stained positive for genomic ZIKV RNA[4, 8, 9, 17]; however, very few spermatozoa containing negative strand ZIKV RNA from these epididymal suspensions were identified. We and others have shown that immature spermatids in the testes are infected by ZIKV. Thus, spermatids infected at a late stage of maturation could harbor and replicate ZIKV RNA, but once spermatids mature into spermatozoa, viral RNA would not be replicated further. This may explain the remnants of Z- staining seen in mature spermatozoa.

The mechanism(s) by which ZIKV is sexually transmitted remain unclear, as the cell(s) that produce infectious ZIKV in seminal fluids are unknown. The cellular component of semen is a mixture of spermatozoa, white blood cells, immature germ cells, and sloughed epithelial cells [18]. Seminal plasma is a mixture of fluids produced mainly by the seminal vesicles and prostate, although the bulbourethral glands and the epididymis also contribute to semen volume [18]. It is unlikely that mature spermatozoa serve as a critical site for generation of infectious virus in semen as they are transcriptionally inactive, devoid of tRNAs and do not contain organelles such as cytoplasmic ribosomes or endoplasmic reticulum, and spermatozoa do not translate protein until post-capacitation [12–15, 19]. Furthermore, vasectomy does not preclude sexual transmission of ZIKV [1, 10]. Human Immunodeficiency virus (HIV), another sexually transmitted virus, has been shown to infect spermatogonia, but not mature spermatozoa [20].

**Fig 11:**
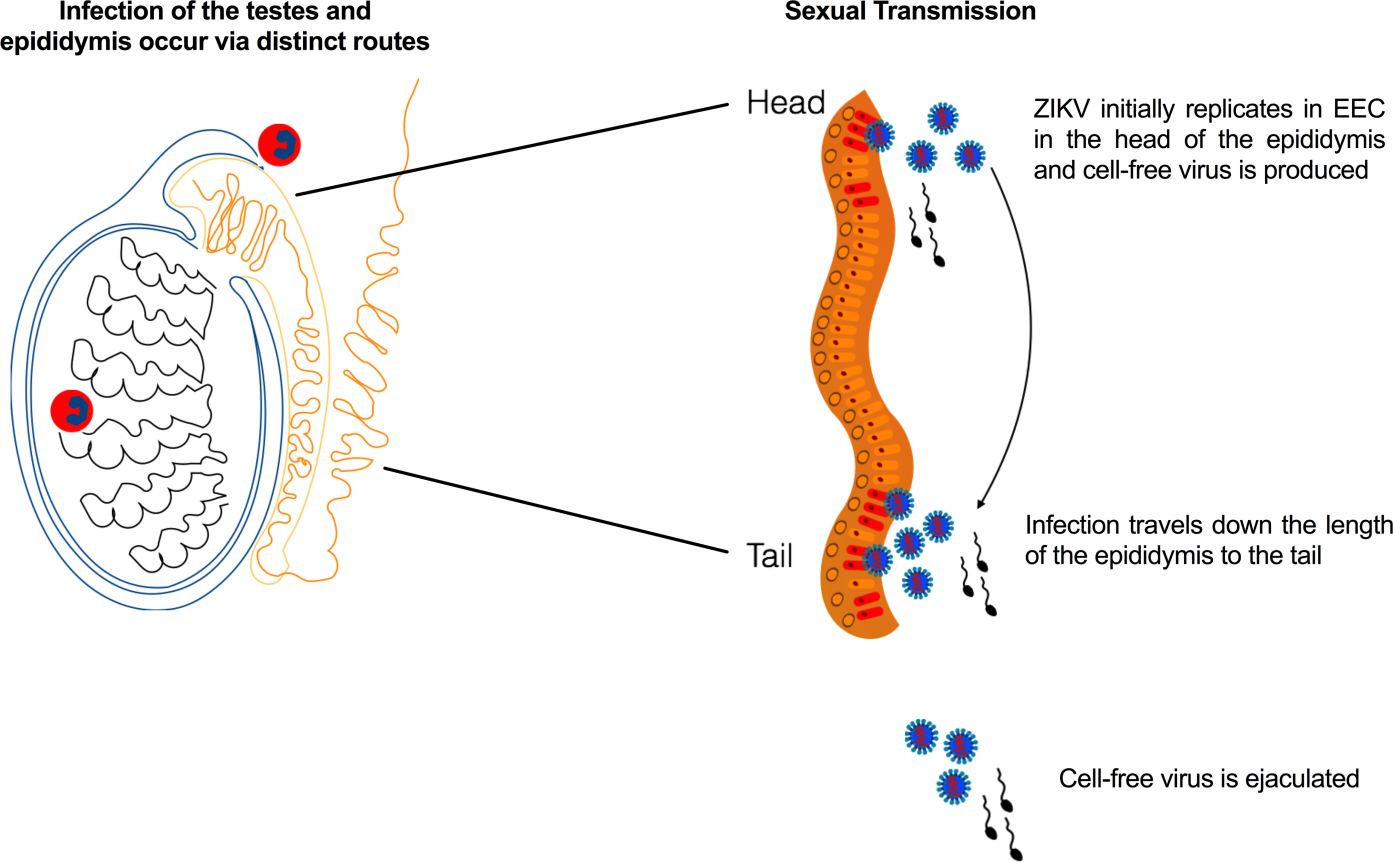
Model of seminal shedding of ZIKV. Tissue ISH suggests that the testes and epididymides are infected concurrently by trafficking leukocytes containing ZIKV. Once leukocytes transport ZIKV to the testis, peritubular myoid cells are the first cell type to become infected, and then the infection spreads to the seminiferous epithelium. In the epididymis, infected epididymal epithelial cells produce viral particles, which then spread intraluminally throughout the epididymis. Cell-free (and cell-associated virus) is ejaculated.

The intense staining for ZIKV RNA (both genomic and replicative) in the head of the epididymis, as compared to the testes, may be a result of the increased susceptibility of the epididymis to leukocyte infiltration. Circulating ZIKV-infected blood cells could initiate infection in the head (caput) of the epididymis by traversing the tubuli recti or rete testis [21]. Tissue ISH identified interstitial inflammation in the caput epididymis, and flow cytometry detected ZIKV-infected infiltrating leukocytes into the epididymal lumen by dpi 8. Thus, leukocytes could be responsible for transferring virus to the epididymal epithelium. By dpi 8, ZIKV genomic and replicative RNA stained most prominently in both intact and necrotic EEC. The ZIKV RNA staining was restricted to the head of the epididymis, and it was not until dpi 10 that the tail of the epididymis demonstrated staining for ZIKV replicative RNA. These data suggest a model (Fig 11): infected EEC in the epididymal head produce viral particles that spread intraluminally throughout the epididymis, to infect the tail and ductus deferens. In support of this model, the majority of infectious virus in the ejaculates was cell-free, and cell-free infectious titers increased over time. Infected EEC and infected leukocytes in the epididymal lumen are most likely the cellular reservoirs producing cell-free infectious virus. Another possibility is that the vas deferens is also infected by a hematogenous route. Circulating white blood cells could initiate infection in the vas deferens, leading to infection of epithelial cells in the vas deferens. Thus, this could be another potential reservoir of ZIKV virions and/or viral RNA and explains the shedding of ZIKV RNA and infectious virus in semen of vasectomized mice [4]. Spermatozoa are not likely to contribute cell-free infectious virus, as they exhibited limited staining for ZIKV genomic RNA and even less staining for replicating ZIKV RNA.

For HIV, the epididymis is one potential reservoir for infectious virus in seminal fluid. Histopathology of the male reproductive tract, from both human autopsies and macaque studies, have identified HIV and Simian Immunodeficiency virus in the epithelium and connective tissue of the epididymis, in adherent myeloid, leukocyte and lymphocyte populations in the epididymis, and in infiltrating leukocytes in the epididymal lumen and stroma [22–26]. Since sexual transmission of HIV occurs via cell-free infectious HIV virions and leukocyte-associated HIV in seminal fluid, these tissue-adherent HIV-infected cells in the epididymis have been identified as a potential source of cell-free infectious HIV[27].

A limitation of this model is that AG129 mice are highly susceptible to ZIKV infection. A less susceptible mouse model that does not show severe morbidity or mortality after ZIKV infection could be developed to assess long-term shedding of ZIKV RNA in seminal fluids. Such a model could also be used to study the potential for recrudescence of infectious ZIKV during long-term shedding. EECs express IFN-inducible antiviral proteins after activation of microbial sensors [28], and it may be this pro-inflammatory and anti-viral state that eventually inhibits the production of infectious virus in semen. Thus, EEC viral growth restriction may be crucial for reducing infectious virus in semen. The contribution of EEC to resolving viral infection and determination of whether the epididymides or testes play a role in persistent viral RNA shedding needs to be assessed.

The data reported here provide critical insight into potential route(s) of sexual transmission following ZIKV infection of the male murine reproductive tract. Identification of the tissue/cellular reservoir(s) of infectious ZIKV is required for better design and employment of therapeutic interventions, assessing the risk assessment of sexual transmissibility and determining the safety of seminal samples used for assisted reproductive technology. It is crucial to determine what cell populations in the male reproductive tract harbor persistent viral infection, and what the effects of immunosuppression are on the recrudescence of infectious virus production in these cell populations. Therapeutic interventions for *in vitro* fertilization treatments for women whose partner is an HIV-infected man include semen washing to separate spermatozoa from leukocytes and other non-spermatozoa cell types before use [29, 30]. Whether similar treatments could be used for men with detectable ZIKV RNA in their semen is unknown; however, these data indicate that the highest prevalence of infectious virus in seminal fluid is associated with EEC production of free vial particles in the tail of the epididymis. Answering the key questions outlined above will provide critical guidance for healthcare providers and patients seeking pre-pregnancy counseling.

## Materials and methods

### Viruses

The virus isolates used in this study were: PRVABC59 (Puerto Rico 2015; Vero passage 3), P6-740 (Malaysia 1966; suckling mouse passage 6, Vero passage 3), and FSS13025 (Cambodia 2010; Vero passage 4).

### Inoculation of AG129 mice

Mice deficient in interferon α/β and -γ receptors (AG129 mice) were bred in-house, and the receptor knockout genotype of the mice was confirmed as described previously [4]. 12-to 18-week-old male mice were inoculated subcutaneously in the rear footpad with 3 log_10_ PFU of ZIKV strain PRVABC59, P6-740, or FSS13025 diluted in PBS. Mice were euthanized after isoflurane-induced deep anesthesia followed by cervical dislocation. Tissues were collected at time of euthanasia and mice were not perfused prior to necropsy. For plaque assays on the testes and seminal vesicles, tissues were weighed and homogenized using a pestle in an equal weight/ volume of BA-1 medium, and then clarified by centrifugation and serially diluted for plaque assay on Vero cells to enumerate titers represented as plaque forming units (PFU per gram tissue). For plaque assays on the epididymides, a portion of the epididymal lumen single cell suspension prepared for flow cytometry was serially diluted for cell plaque assay to enumerate infectious titer (PFU per epididymides). The overlay for the plaque assay was added four days post-inoculation. A portion of the epididymal cell suspension was also used to enumerate mature spermatozoa. The cells were stained with trypan blue to increase contrast and observed under bright field microscopy. Intact, mature spermatozoa were counted on a hemocytometer.

### Collection of seminal fluids from male AG129 mice

Seminal fluids from male AG129 mice were collected as previously described [4, 5]. In brief, inoculated male mice were housed individually, and each evening (beginning on dpi 5) three to five female CD-1 mice were introduced into the cage. The following morning, mating activity was determined by the presence of a copulatory plug in the female. Mated females were euthanized by isoflurane anesthesia followed by cervical dislocation. 500 μL of BA-1 media was used to gavage both horns of the uterus. Infectious ZIKV in the seminal fluids was titrated by Vero cell plaque assay as described above (PFU per mL).

To separate the ejaculates into cell pellet and seminal plasma fractions, ejaculates were spun at a 250 rcf, and the supernatant was diluted and filtered through a 0.2 μM filter to remove any remaining cells. The remaining cell pellet was washed three times with PBS and subsequent centrifugations to remove any extracellular virions before being sheared with a needle to release intracellular virions. Infectious ZIKV was titrated by Vero cell plaque assay.

### Antibodies, flow cytometry staining and PrimeFlow RNA assay

Single-cell suspensions of the testis and epididymides were generated by mechanical disruption. Disruption of the testis involved decapsulating each testis and homogenizing in 1X PBS and filtering the cells through a 70 μM filter (Sigma) followed by multiple washes of the filter with 1X PBS. The epididymides were minced in 1 mL of 1X PBS and incubated at 37°C for 10 minutes. The resulting cell suspension was filtered through a 70 μM filter (Sigma).

For RNA staining analyses, cells were treated as per manufacturer’s instructions using a commercially available kit (PrimeFlow RNA Assay, Thermo Fisher Scientific) and a probe set targeting either positive or negative strand ZIKV RNA (designed and synthesized by Thermo Fisher Scientific; Catalog VF-19981 ZIKV Polyprotein Type 1, VF4-20142 ZIKV Polyprotein Type 4, VF1-20258 ZIKV Polyprotein negative strand Type 1). Briefly, cells were stained with fluorochrome-conjugated mAb against mouse anti-CD45 (clone 30-F11, Thermo Fisher Scientific) for 30 minutes at 4°C, and subsequently cells were fixed, permeabilized and stained with the ZIKV RNA, or PRM2 probe sets. Cells were collected at low pressure on an Accuri C6 flow cytometer (BD Biosciences) equipped with blue (488 nm) and red (635 nm) lasers. Single stained samples, UltraComp eBeads microspheres (cat. 01–2222, Thermo Fisher Scientific) stained with Alexa Fluor 488 or 647 RNA compensation controls or mouse anti-CD45 PE-Cy7, were used for compensation controls. FlowJo10.2 (Tree Star, Inc., Ashland, OR) was used to analyze data, including the FlowJo automated compensation procedure. Gates for viral RNA positive cells were set using single cells suspensions from uninoculated mice that underwent the same staining procedures. Gates for ZIKV RNA staining were set with less than 0.2% background in uninoculated mice. See S1 Fig for the entire gating strategy. As a control for the PrimeFlow RNA Assay, a spleen from an uninoculated mouse was isolated on each day cells were prepared for flow cytometry and stained for a positive housekeeping probe set (probes targeting GAPDH, PIPB and β-actin mRNAs). Greater than 90% of live splenic cells stained positive for the positive control probe set on each day of staining (see S2 Fig).

### Fluorescence microscopy of mature spermatozoa

10 μL aliquots of the epididymis single cell suspensions processed for PrimeFlow and stained with ZIKV RNA probes were dried onto a microscope slide. ProLong Diamond Antifade mountant was used to mount coverslips to the samples. Slides were visualized on a Zeiss LSM 800 confocal laser-scanning microscope using the 63x objective. Mature spermatozoa were counted as follows: sperm had to be clearly distinguished from one another, and only mature spermatozoa with a head attached to a midpiece and tail were counted. 50–100 sperm were counted for two mice from each ZIKV-inoculated group.

### Tissue RNA *In Situ* hybridization

Tissues were placed into 10% neutral buffered formalin for 3 days and then stored in 70% ethanol prior to processing. Sections were cut at 4 microns and stained by *in situ* hybridization (ISH). The ViewRNA ISH tissue assay (ThermoFisher) with probe sets targeting either positive or negative strand ZIKV RNA or murine PRM2 mRNA (GenBank accession number X07626.1) were utilized. As a negative control, tissue sections from an uninfected male mouse were stained using the ZIKV (+) and (-) probe sets. As a positive control, tissues were stained using probes against the housekeeping murine mRNAs GAPDH, PIPB, and β-actin. Tissue sections were de-paraffinized and underwent an optimized View RNA ISH tissue assay, with pre-treatment and protease treatments lasting ten minutes each. Positive staining (red punctate staining) was detected using an alkaline-phosphatase label probe. Nuclei were counterstained with Gill’s Hematoxylin I.

### Statistical analyses

ZIKV titers were compared using 2way ANOVA. The percent of Z+ and Z- cells were compared using 2way ANOVA or t-test. All tests were performed in Prism7.

### Animal ethics statement

All experiments involving mice were approved by institutional animal care and use committee (IACUC) at the Division of Vector-Borne Diseases, Centers for Disease Control and Prevention (Protocol 16–013). All protocols and practices for the handling and manipulation of mice were in accordance with the guidelines of the American Veterinary Medical Association (AVMA) for humane treatment of laboratory animals.

## Supporting information

S1 Fig Mature spermatozoa and epididymal luminal cells staining positive for ZIKV RNA. Epididymal lumen cell suspensions were prepared and stained as described in the methods. An aliquot of the suspension was spun onto a glass microscope slide and a coverslip mounted using ProLong Diamond Anti-fade mountant. (A-C) are representative confocal images of epididymal lumen cells from ZIKV-inoculated mice. (A) Cells were labeled with an Alexa Fluor 488 label probe specific for the ZIKV RNA (+) strand. We observed many foci of ZIKV RNA (green) in round cells from the cell suspensions. Very little staining was observed in spermatozoa. (B) Cells were co-labeled with an Alexa Fluor 488 label probe specific for the ZIKV RNA (+) strand and with an Alexa Fluor 647 label probe specific for the ZIKV RNA (-) strand. Once again, the majority of staining for the ZIKV RNA (+) (green) and (-) (magenta) strands occurred in round cells. Very few spermatozoa stained positive for either the ZIKV RNA (+) or (-) strands. When staining was seen in spermatozoa, the foci were small and dim.
(EPS)

S2 Fig Flow cytometry gating scheme to identify CD45+ leukocytes and ZIKV RNA (+) cells. Testis and epididymides were harvested from 18–20 week-old AG129 male mice. Single testis cells and epididymis cells suspensions were prepared and stained as described in the methods. A gate to exclude debris was set first (1), followed by a gate to exclude aggregates (2). A Time vs. FSC-A gate was applied next (3). This gate is important to get rid of artifacts that occur when the cytometer pressurizes and de-pressurizes at the start and end of each run. If a live-dead stain was used, a gate for live cells was applied next (4). Since the PE channel was unused, any positive events in this region are not valid, and so a gate was set to exclude any PE+ events (5). This population was then analyzed for CD45 expression (x-axis) and ZIKV RNA events (y-axis). The ZIKV RNA+ events gate was set using an uninfected control mouse (6).
(EPS)

S3 Fig Splenic control to validate RNA flow cytometry staining. Spleens were harvested from 18–20 week old AG129 mice. A single cell suspension of the spleen was prepared and stained as described in the methods. The probe set for murine housekeeping mRNAs (a blend of probes directed against GAPDH, β-actin and PIPB) were used for staining. This control was carried out each time the testis and epididymis single cells suspensions were stained with the ZIKV RNA probe sets. The splenic samples were gated as described in S1 Fig. On average, 91.1% (Std dev 5.8%) of live splenic cells stained positive for the housekeeping probe set.
(EPS)
